# Artificial Autopolyploidization Modifies the Tricarboxylic Acid Cycle and GABA Shunt in *Arabidopsis thaliana* Col-0

**DOI:** 10.1038/srep26515

**Published:** 2016-05-23

**Authors:** Fredd Vergara, Jun Kikuchi, Christian Breuer

**Affiliations:** 1 Environmental Metabolic Analysis Research Team, CSRS, RIKEN. 1-7-22 Suehiro-cho, Tsurumi-ku, Kanagawa-ken, 230-0045, Japan; 2 Graduate School of Medical Life Science, Yokohama City University, 1-7-29 Suehiro-cho, Tsurumi-ku, Kanagawa 230-0045, Japan; 3 Graduate School of Bioagricultural Sciences, Nagoya University, 1 Furo-cho, Chikusa-ku, Nagoya, Aichi 464-0810, Japan; 4 Cell Function Research Team, CSRS, RIKEN. 1-7-22 Suehiro-cho, Tsurumi-ku, Kanagawa-ken, 230-0045, Japan.

## Abstract

Autopolyploidy is a process whereby the chromosome set is multiplied and it is a common phenomenon in angiosperms. Autopolyploidy is thought to be an important evolutionary force that has led to the formation of new plant species. Despite its relevance, the consequences of autopolyploidy in plant metabolism are poorly understood. This study compares the metabolic profiles of natural diploids and artificial autotetraploids of *Arabidopsis thaliana* Col-0. Different physiological parameters are compared between diploids and autotetraploids using nuclear magnetic resonance (NMR), elemental analysis (carbon:nitrogen balance) and quantitative real-time PCR (qRT-PCR). The main difference between diploid and autotetraploid *A*. *thaliana* Col-0 is observed in the concentration of metabolites related to the tricarboxylic acid cycle (TCA) and γ-amino butyric acid (GABA) shunt, as shown by multivariate statistical analysis of NMR spectra. qRT-PCR shows that genes related to the TCA and GABA shunt are also differentially expressed between diploids and autotetraploids following similar trends as their corresponding metabolites. Solid evidence is presented to demonstrate that autopolyploidy influences core plant metabolic processes.

A key realization of plant evolutionary genomics is that independent of their present ploidy status, all modern flowering plant genomes derive from repeated, episodic events of whole-genome duplication -polyploidization-1. Polyploidy is defined as the possession of three or more complete sets of chromosomes and it is estimated that 47% to 70% of all living angiosperms are polyploids^2^. Polyploidy can be classified into allopolyploidy, which is the combination of two or more sets of distinct genomes (species hybridization), and autopolyploidy, with multiple chromosome sets derived from a single species^3^. A special case of autopolyploidy is whole-genome duplication, which leads to a 2-fold increase in the amount of nuclear DNA. Among angiosperms, most eudicot plants have recently been argued to be descendants of an ancient hexaploid ancestor^4^ and in the case of the genus *Arabidopsis* phylogenomic analysis shows two further whole-genome duplications^5^. Thus, a gene that was single-copy in an ancestral angiosperm about 200 million years ago could, in principle, have turned into a 12-member family in *Arabidopsis* by means of polyploidization alone^6^,^7^. Although polyploidy is widespread in plants much remains unknown about how duplicate genes and genomes function in the early stages of polyploidization (neopolyploidization). Furthermore, it is unclear how the duplicate genes diverge in function during plant evolution but the common occurrence of polyploidy suggests an evolutionary advantage of having multiple sets of genetic material for adaptive evolution^3^. Autopolyploidization could, on the other hand, represent a disadvantage because it might interfere with proper chromosome segregation^8^,^9^, yet the existence of naturally occurring autopolyploids suggests that plant cell metabolism can adjust to the increased number of chromosome copies and its effect on gene dosage.

Marked effects have been observed on gene expression as a consequence of alloploidization^10^. Allopolyploids of *Arabidopsis thaliana* have shown different levels of gene expression^11^,^12^,^13^, DNA methylation^14^, proteomic pattern^15^ and starch, sugar and chlorophyll concentrations^16^. Allopolyploids of the related species *Brassica napus* varied in flowering time^17^, DNA methylation, seed set and leaf morphology^18^ and proteomic profile^19^. Thus, the effect of allopolyploidization on plant physiology has been documented; however, no systematic study has been reported aiming to identify the metabolic differences produced by autopolyploidization in plants even if empirical estimates show that autopolyploidy and allopolyploidy frequencies are comparable among vascular plant species^20^. The present study addresses this important question in plant biology by comparing the physiology of diploid and artificially produced autotetraploids of *A*. *thaliana* Col-0 using diverse analytical chemical and molecular biological techniques. Nuclear magnetic resonance (NMR) and carbon:nitrogen (C:N) elemental analyses were used to provide metabolic profiles of diploid and autotetraploid individuals. A metabolomics approach based on ^1^H NMR spectra was used to highlight differences in chemical composition between diploids and autotetraploids; and comparative analysis of 2D NMR spectra, to propose candidate compounds with the largest contributions to the statistical variability between ploidies. Elemental analysis was conducted to understand differences in C:N balance between ploidies. Using the information generated with the metabolomics study, quantitative real-time PCR was performed on selected genes whose metabolic products showed the largest NMR variability between ploidies. Our findings show that the metabolism of *A*. *thaliana* Col-0 displays strong differences correlated with the plant’s ploidy level.

## Results

**Figure 1:**
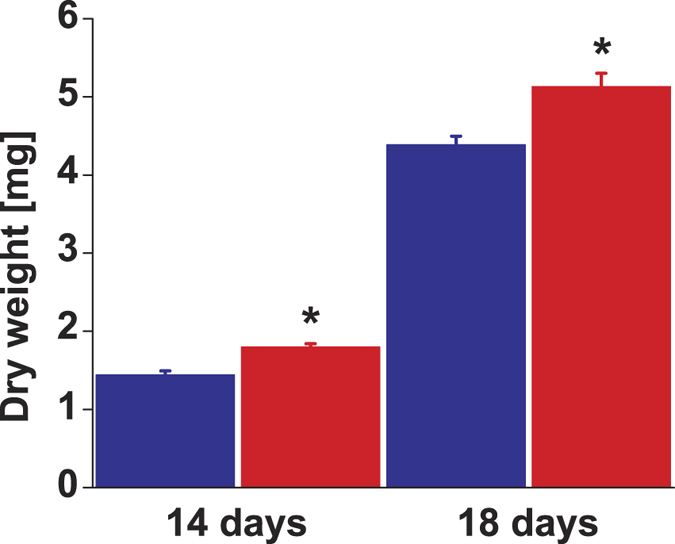
Dry weight of *Arabidopsis thaliana* Col-0 above-ground tissue at 2 different ages. Diploids: blue bars, autotetraploids: red bars. Bars represent means and standard error of the means of 30 plants. *t*-test showed significant differences between ploidies (*P < 0.05).

**Figure 2:**
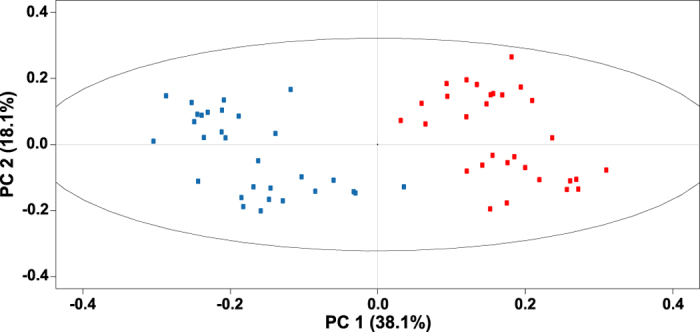
PCA score plot of ^1^H NMR spectra of *Arabidopsis thaliana* Col-0 whole above-ground tissue. Metabolic profiles of 14-day-old diploids (blue circles) and autotetraploids (red circles) are represented. Each dot represents the metabolic profile of a single plant. Each ploidy had a clearly distinct NMR-based metabolic profile as no overlap is observed between diploids and autotetraploids.

**Figure 3:**
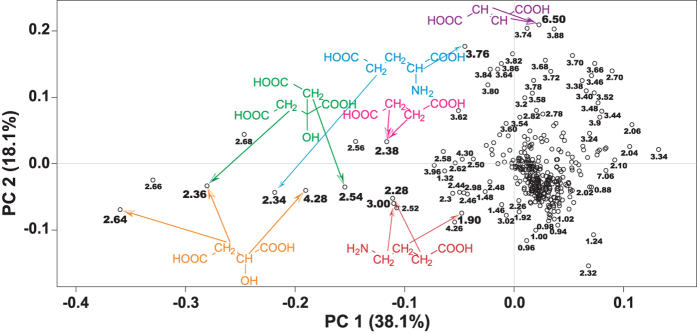
PCA loading plot of ^1^H NMR spectra of 14-day-old *Arabidopsis thaliana* Col-0 whole above-ground tissue. Compound identification of the most variable signals was performed by comparing corresponding ^1^H-^13^C HSQC signals with a database of authentic standards. The shown compounds are intermediates of the tricarboxylic acid cycle -TCA- (malate (orange), citrate (green), succinate (pink), and fumarate (purple)) or are involved in carbon:nitrogen balance (γ-aminobutyric acid (red) and glutamate (blue)).

**Figure 4:**
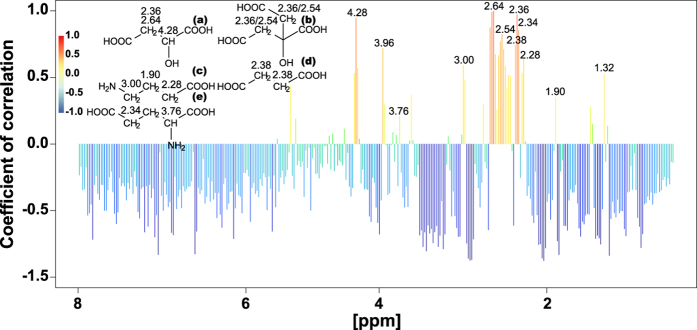
1D-STOCSY of *Arabidopsis thaliana* Col-0 whole above-ground tissue. 14-day-old diploid and autotetraploid ^1^H NMR spectra were used for correlation. Driver peak: 2.64 (see experimental procedures). All compounds showing statistical correlation (signals displayed in red gradient) participate in the tricarboxylic acid cycle -TCA- (malate (a), citrate (b), and succinate (d)) or carbon:nitrogen balance (γ-aminobutyric acid (c), and glutamate (e)).

*Arabidopsis thaliana* Col-0 diploids and autotetraploids analyzed at two ages (14- and 18-days-old) showed the same trends independent of their age. Thus analytical chemical and molecular biological results are shown only for 14-day-old plants. Colchicine-induced autopolyploidization of *A*. *thaliana* diploids was confirmed by the doubling in the amount of DNA for every peak detected in flow cytometry (Fig. S1). Multiple peaks are detected as a consequence of endoreduplication. Autotetraploids accumulated more biomass than diploids (Fig. 1). Diploid and autotetraploid *A*. *thaliana* above-ground tissue had a strongly different ^1^H NMR metabolic profile as evidenced by the formation of separated clusters for each ploidy on the PCA score plot (Fig. 2). The loading plot showed that a group of highly variable bins aligned along PC1 (Fig. 3). 1D-STOCSY using 2.64 as driver peak, the most variable bin according to the loading plot, showed that bin 2.64 had a strong positive correlation with bins 2.36, 2.54 and 4.28 (Fig. 4). Signals at 2.36, 2.64 and 4.28 ppm on the ^1^H dimension of the ^1^H-^13^C HSQC spectrum of *A*. *thaliana* mixed extracts showed cross peaks on the ^13^C dimension at 45.40, 45.40 and 73.20 ppm, respectively. These signals correspond to malate in our database of authentic standards. Signals in the sample at 2.54 and 2.64 (^1^H) showed cross peaks at 48.45 and 48.45 ppm (^13^C). These signals matched with citrate in our database. Bin 2.64 was also positively correlated with bins 1.90, 2.28 and 3.00. These signals did not show cross peaks in the ^1^H-^13^C HSQC spectrum but they matched well with signals in our ^1^H NMR database for γ-aminobutyric acid. Additional strong positive correlation was observed between bin 2.64 and bin 2.38, signal that corresponds to succinate in our database. Bin 2.64 correlated too with bins 2.34 and 3.76, and these signals showed cross peaks with ^13^C signals at 36.25 and 57.05 ppm corresponding in our database with glutamate. Other bins showing high variability according to the loadings plot were bin 6.50, which showed a cross peak at 138.20 (^13^C) that corresponds to fumarate in our database. All the identified metabolites participate in the tricarboxylic acid and GABA pathways. Other bins showing high variability in the loadings plot correspond to sugars. In the 1D-STOCSY bin 2.64 was not correlated with bin 6.50 but sugar signals were negatively correlated.

**Figure 5:**
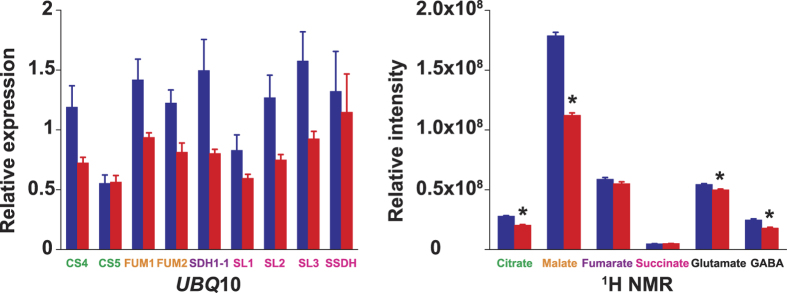
Relative gene expression and metabolite concentration in *Arabidopsis thaliana* Col-0 above-ground tissue. Graphs show quantitative real-time gene expression (a and b) and ^1^H NMR signal intensity (c) of enzymes and metabolites participating in the tricarboxylic acid cycle (TCA) and carbon:nitrogen balance (GABA shunt). Diploids: blue bars, autotetraploids: red bars. Bars represent means and standard error of the means of 8 plants (qRT-PCR) or 30 plants (^1^H NMR). Metabolites showing the largest variation according to principal component analysis of ^1^H NMR spectra and their corresponding genes are compared between ploidies. Actin 2 and ubiquitin 10 expression levels were used for normalizing qRT-PCR values. *t*-tests were performed to identify significant differences between ploidies (*P < 0.05). Names of genes and their corresponding metabolites are displayed in the same color.

**Figure 6:**
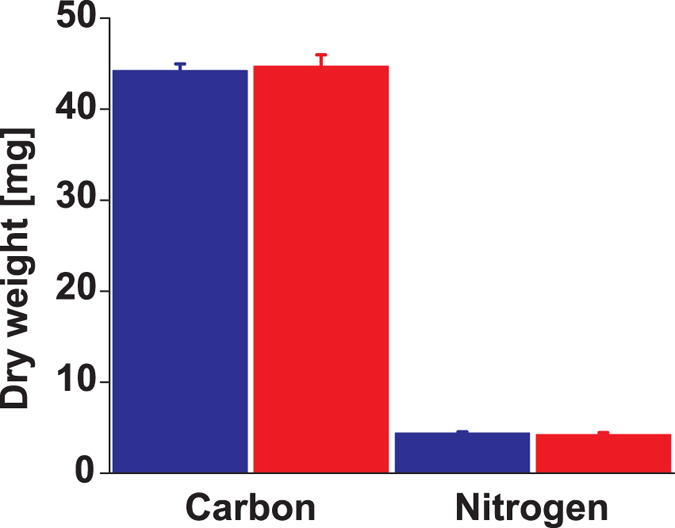
Carbon:nitrogen elemental analysis in *Arabidopsis thaliana* above-ground tissue at 14-day-old. Bars represent means and standard error of the means of 10 plants. No statistic differences were found between ploidies. Diploids: blue bars, autotetraploids: red bars.

Gene expression levels showed comparable trends independent of the normalizing gene used, *UBQ*10 or *ACT*2. qRT-PCR analysis did not reveal any statistically significant difference for the expression of any of the studied genes between ploidies (Fig. 5a, Fig. S2). However, the mean values for most of the genes were higher in diploids, a tendency also observed for the ^1^H NMR signal intensities of the corresponding metabolites (Fig. 5b). No difference was detected in the carbon:nitrogen ratios between ploidies (Fig. 6).

## Discussion

Gene duplication is a powerful evolutionary force as it allows genetic novelty resulting from gene neofunctionalization^21^,^22^. If a gene is duplicated, then at least one of the two genes can be mutationally altered and survive purifying selection (because the mutant gene is covered)^23^. Autopolyploidization represents the most extreme case of gene duplication as all the genes in an organism are duplicated (whole-genome duplication) and in evolutionary time it can lead to speciation through the mechanism of reciprocal gene loss^24^. Recently formed autopolyploids are poised with immediate challenges connected with meiosis, *e*.*g*., restoring chromosome pairing and the establishment of a breeding population^25^. For example, *Arabidopsis arenosa* (a close relative of *A*. *thaliana*) is known to naturally exist as diploid and autotetraploid cytotypes and genome-wide data showed how natural selection has acted on meoisis genes^26^. Apart from these cytological challenges, neopolyploids also have to cope with the challenges imposed by the environment. Differences in the anatomy and in the timing of events in the life cycle could affect competition, environmental tolerances, and reproductive isolation of polyploids^27^. A well-known effect of autopolyploidization is an increase in cell and organ size^27^,^28^, as reflected in the bigger size of the autotetrapolyploid *Arabidopsis thaliana* Col-0 used in this study (Fig. 1).

Another crucial trait that deeply influences plant ecology is plant metabolism^29^,^30^,^31^. It is in this particular aspect of plant autopolyploidy that the present study pioneers. It presents the first evidence on how autopolyploidization modifies plant metabolism. Metabolites are the final expression of genes and about a century ago the formulation of the gene balance hypothesis implied that ‘gene products [metabolites] interact with set stoichiometries and there is a tendency for purifying selection to not recognize whole-genome duplications because all genes in the genome change dosage contemporaneously, so balances are unchanged’^23^,^32^. The NMR results in this study contradict this notion as the variation in metabolite concentration is compound specific (Fig. 5b) and thus stoichiometries for the same gene products are different in diploids and autotetraploids. Moreover, variation in transcript levels is also gene product specific (Fig. 5a, Fig. S2). Previous reports show that autotetraploid *A*. *thaliana* Col-0 have an increased number of chloroplasts per cell as diploids^33^ but it is not known if the higher chloroplast density modifies photosynthetic rates in autotetraploids. Chloroplast and mitochondrial metabolisms are connected via metabolites of the tricarboxylic acid cycle -TCA-34. TCA metabolites identified in this study vary between diploids and autotetraploids (Figs 2 and 3). These metabolites also participate in cell guard metabolism and regulators of stomatal opening^35^. This opens the possibility that differences might exist between diploids and autotetraploids in terms of gas exchange, which in turn might be connected to different photosynthetic rates produced by the different chloroplast density. Among all the TCA metabolites varying between ploidies, malate displays the largest difference between diploids and autotetraploids. Functional roles for malate in plants are quite diverse including, but not limited to: respiration and energy generation, photosynthesis (both C3 and C4), fatty acid oxidation, lignin biosynthesis, nitrogen fixation and amino acid biosynthesis, ion balance, uptake of phosphorus and iron, aluminum tolerance and pulvinal and stomatal function^36^. Root excretion of malate has been associated with aluminum tolerance, phosphate nutrition and the establishment of microbial communities. *A*. *thaliana* Col-0 autotetraploids also have a lower concentration of glutamate and γ-amino butyric acid (GABA), both amino acids known to participate in the maintenance of carbon:nitrogen (C:N) balance^37^,^38^,^39^. The results of the present study show, however, that the differences in concentration for glutamate and GABA between diploids and autotetraploids do not affect the C:N balance regardless of the ploidy (Fig. 6). Glutamate and GABA are connected to the TCA cycle via a short pathway composed of three enzymes called the GABA shunt because it bypasses two steps of the TCA cycle. Remarkably, statistical correlations of the NMR spectra show that both TCA and GABA metabolites vary coordinately within plants (Fig. 4) suggesting that entire metabolic pathways are down regulated in the autotetraploids.

Interestingly, diploid and autotetraploid populations of *A*. *arenosa* are mostly geographically separated^40^. Similar geographic segregations have been observed in other multiploid plant species^41^,^42^,^43^. This allopatric distribution can be the result of reproductive incompatibilities between ploidies and has profound consequences for the ecology of plants. In the specific case of plant-insect interactions, differences have been detected in the insect species composition and extent of herbivory and pollination experienced by diploid and autotetraploid plants^44^,^45^,^46^,^47^. Unfortunately, these studies did not analyze volatile organic or defensive compounds, molecules known to regulate pollination and herbivory. The precursors of these specialized metabolites are produced by core metabolic pathways like the ones shown in this study to be modified by autopolyploidization in *Arabidopsis thaliana*. This fact opens the possibility that concentrations of specialized metabolites could also be different in individuals of different ploidy levels in multiploid plant species.

In summary, the metabolic differences produced by autopolyplody can have profound effects for the development and ecological interactions of plant neopolyploids. With at least 24 ancient autopolyploidization events identified to have occurred during the evolution of vascular plants^48^ it is completely relevant to have a clearer picture of how this mutation can affect the fitness of the newly formed autopolyploids. The prevalent view is that autopolyploidy is a common and important element of plant diversity. This study suggests how divergent ecological interactions might arise from the physiological differences existing in neoautotetraploids and the ecological differences may in turn lead to differential evolutionary paths for autopolyploid plants.

## Materials and Methods

### Plant material

*Arabidopsis thaliana* Col-0 diploid plants were treated with colchicine as previously described to obtain autotetraploid seeds^49^. Diploid and autotetraploid seeds were sown in vermiculite and plants were kept at 25 °C and 50% relative humidity with 24 h light (75 μmol m^−2^ s^−1^) and plenty of water. Plants were harvested 14 and 18 days after sowing. For each plant the whole above ground tissue was collected. Fresh tissue was used for flow cytometry. For all other analyses harvested plants were immediately flash-frozen with liquid nitrogen. Water was removed with a freeze-dryer and samples were immediately processed for chemical and molecular biological analyses.

### Flow cytometry

Cell nuclei were extracted and stained simultaneously using the following suspension based on Galbraith buffer: Tris (242 mg, Wako Chem.), MgCl_2_ · 6H_2_O (8.13 mg, Wako Chem.), Triton X 100 (10 μl, Alfa Aesar) DAPI Cellstain^®^ solution (40 μl, Dojindo); milliQ water was added up to 10 ml and pH was adjusted to 7.5 with HCl. Plants were placed on a Petri dish and covered with 500 μl of the extracting/staining suspension. Plants were then chopped with a razor blade and the suspension containing the free cell nuclei filtrated with CellTrics^®^ filters (30 μm, Partec). The filtrate was collected in a plastic tube, gently mixed and injected into the flow cytometer. A Partec PA flow cytometer was operated using 0.02% Triton as sheath fluid at a flow rate of 1 μl s^−1^ with a par gain of 290. Data were acquired in lg1 mode. The FCS files containing the flow cytometry data were converted to ASCII files using the software LDATAPP [http://www.cyto.purdue.edu/flowcyt/software/ldatapp.htm]. ASCII files were plotted with Origin^®^ 7 SR1 [http://www.originlab.com] as a continuous function and curves smoothed with an adjacent averaging algorithm included in the software.

### Sample preparation for NMR

The freeze-dried above-ground tissue of 30 diploid and 30 autotetraploid plants were weighed and its polar compounds extracted by grounding the plant material with a plastic pestle and an 1.5 ml Eppendorf tube filled with 700 μl of 1 mM KPi (10.7 mg K_2_HPO_4_ + 5.24 mg KH_2_PO_4_) in D_2_O (D 99.9%, Cambridge Isotope) containing 1 mM DSS-*d*6 (D 98%, Cambridge Isotope) as internal standard. The samples were centrifuged at 17,500 *g* at 25 °C for 10 min and 600 μl of the supernatant recovered. Due to differences in mass of individual plants, dilutions were performed against the smallest plant in each sampling time in order to normalize the amount of material under analysis. The final per sample volume was 600 μl of deuterated solvent.

### NMR spectra acquisition and processing parameters

Spectra were recorded using an Avance II 700 Bruker spectrometer equipped with a 5 mm inverse cryoprobe operating at 700.153 MHz for ^1^H and 176.061 MHz for ^13^C. Acquisition temperature was 298 K. ^1^H spectra were acquired using a water suppression pulse program with the following conditions: 0.29 Hz point^−1^, acquisition time = 1.67 s, relaxation delay = 2.5 s and 90° pulse width = 10 μs. 128 transients with 4 dummies were recorded per spectrum. FIDs were Fourier-transformed using no line broadening (LB = 0.0 Hz). The resulting spectra were manually phased and baseline-corrected and calibrated to the internal standard (DSS-*d*6). ^1^H-^13^C HSQC spectra were acquired using the Bruker pulse program hsqcetgp for echo/antiecho gradient with the following conditions: relaxation delay = 1.5 s and 26,410 Hz spectral width in f1 and 9,803 Hz in f2. Qsine (SSB = 2.0) was used for the window function. 16 dummies and 48 transients were collected. FID was Fourier-transformed and the resulting spectrum was manually phased and baseline corrected and calibrated using the internal standard (DSS-*d*6).

### Statistical analysis of ^1^H NMR

Phased and baseline-corrected ^1^H NMR spectra were binned from 8.0 to 0.5 ppm with a bin width of 0.02 ppm using the software automics^50^. Binning was performed applying the total scaling function of automics and the region from 5.0 to 4.6 ppm (HDO signal) was not binned. The matrix with the data of all the binned spectra (355 bins per spectrum) was exported to the software R and processed with the package muma for NMR metabolomics^51^. Principal component analysis (PCA) and 1D-statistical total correlation spectroscopy (1D-STOCSY) analysis were performed according to muma’s manual and applying the following parameters: scaling = “pareto”, imput = “mean”, normalize = TRUE. Batch spectral integration of selected regions of interest (ROI) was performed on ^1^H NMR spectra using rNMR^52^. The next ROIs (expressed in ppm) were used: 2.570–2.547 (citrate), 2.680–2.635 (malate), 6.513–6.500 (fumarate), 2.398–2.389 (succinate), 3.771–3.741 (glutamate) and 3.020–2.987 (γ-aminobutyric acid). *t*-tests were performed on single ROIs in order to identify significant signal intensity differences between ploidies. 1D-STOCSY was applied to the binned data in order to generate a pseudo-NMR spectrum displaying the correlation among the intensities of various peaks across the whole dataset, thus taking advantage of the multi-colinearity of the intensity variables in a set of spectra^53^.

### Candidate compounds based on 2D NMR

In order to increase signal intensity 15 diploid and 15 autotetraploid 14-day-old *A*. *thaliana* extracts were mixed, solvent freeze-dried and sample reconstituted in D_2_O. Coordinates for the resulting ^1^H-^13^C HSQC signals were automatically picked up in Topspin (Bruker). The resulting list of 32 ^1^H-^13^C cross peaks was imported into the software spinassign [http://prime.psc.riken.jp] for signal matching against a database of standard compounds.

### qRT-PCR analysis

Quantitative real-time PCR was performed on the freeze-dried above-ground tissue. RNA from 2 plants was pooled to form 1 sample, and 4 samples per ploidy were analyzed (8 plants in total per ploidy). Conditions for mRNA extraction and gene expression analyses were as previously described^54^. Actin 2 and ubiquitin 10 mRNA concentrations were determined for each sample to normalize expression of individual genes. Oligonucleotides used are shown in the supporting information section (Table S1). *t*-tests were performed on single genes in order to identify significant expression level differences between ploidies.

### Elemental analysis

Total carbon and nitrogen were determined on freeze-dried above-ground tissue using a vario MICRO cube elemental analyzer (Elementar). 10 diploid and 10 autotetraploid plants were used. Plant material was grounded and 1 mg of powder per plant was mixed with 1 mg WO_3_, placed on a Sn capsule and pressed to form a homogeneous pellet. The temperatures of the combustion and reduction tubes were set at 950 °C and 550 °C, respectively. Sulfanilamide was used according to vendor’s instructions as data calibrator.

## Additional Information

**How to cite this article**: Vergara, F. *et al*. Artificial Autopolyploidization Modifies the Tricarboxylic Acid Cycle and GABA Shunt in *Arabidopsis thaliana* Col-0. *Sci. Rep*. **6**, 26515; doi: 10.1038/srep26515 (2016).

## Supplementary Material

Supplementary Information
