# Supplementary material for: Artificial Autopolyploidization Modifies the Tricarboxylic Acid Cycle and GABA Shunt in Arabidopsis thaliana Col-0
Source: Sci Rep. 2016 May 23;6:26515. doi: 10.1038/srep26515 (PMC4876396; doi:10.1038/srep26515)
Supplement: Supplementary Information [file srep26515-s1.pdf]

Supplementary information for the manuscript:

# Artificial Autopolyploidization Modifies the Tricarboxylic Acid Cycle and GABA Shunt in *Arabidopsis thaliana* Col-0

Fredd Vergara[1*], Jun Kikuchi[1,3,4], Christian Breuer[2]

[1]Environmental Metabolic Analysis Research Team, CSRS, RIKEN. 1-7-22 Suehiro-cho, Tsurumi-ku, Kanagawa-ken, 230-0045, Japan; [2]Cell Function Research Team, CSRS, RIKEN; [3]Graduate School of Medical Life Science, Yokohama City University; [4]Graduate School of Bioagricultural Sciences, Nagoya University

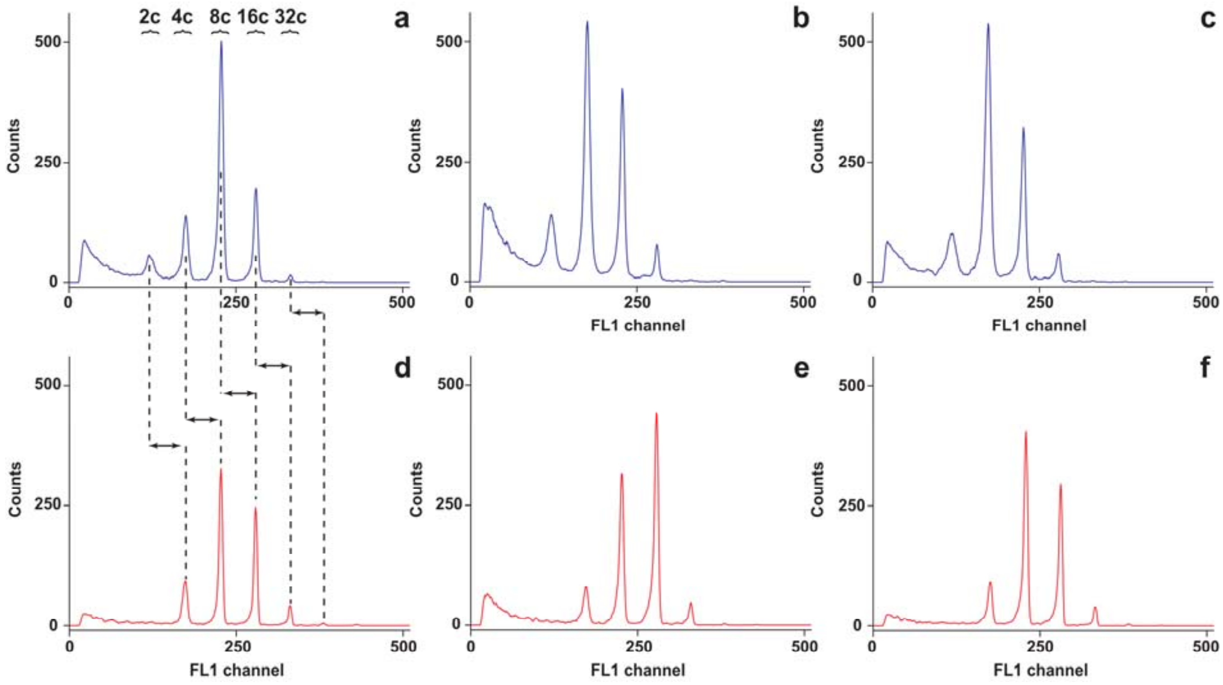


**Figure S1**. Ploidy level determination. Flow cytometry profiles of three randomly chosen diploid (a-c, blue) and autotetraploid (d-f, red) *Arabidopsis thaliana* col-0. Autotetraploidy is clearly observed as a shift in the location of the peaks between the upper and lower graphs across the FL1-axis (2c to 4c, 4c to 8c and so forth), which represents DNA content as a function of fluorescence.

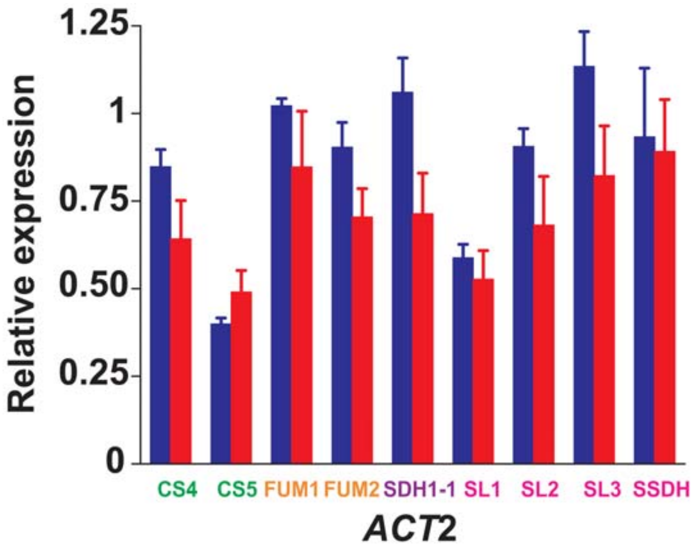


**Figure S2**. Relative gene expression in *Arabidopsis thaliana* Col-0 above-ground tissue. Graph shows quantitative real-time gene expression of enzymes and participating in the tricarboxylicacid cycle (TCA) and carbon:nitrogen balance. Diploids: blue bars, autotetraploids: red bars. Bars represent means and standard deviations of 8 plants (qRT-PCR) or 30 plants ($^{1}$H NMR). Actin 2 expression level was used for normalizing qRT-PCR values. Names of genes are colored as their corresponding metabolites (Fig. 5 in main text).

| Primer name | Primer sequence (5’–3’) | Amplicon region |
|---|---|---|
| CS4*F* | AACCTGATTGCTCGTGTTCC | Citrate synthase |
| CS4*R* | GCCTCATGAGCTCTTTCACC | Citrate synthase |
| CS5*F* | AACAGTGGCAAGGTTGTTCC | Citrate synthase |
| CS5*R* | CCACGGGTTCTTGACCTTT | Citrate synthase |
| FUM1*F* | GGTTTGTGCACAGGTTATGG | Fumarase |
| FUM1*R* | TTTTTCTCGAACGAAGCTGAA | Fumarase |
| FUM2*F* | GCAAGCGCTCTCTTACATTC | Fumarase |
| FUM2*R* | TGAGCTCTTTTGGCTACTGCT | Fumarase |
| SDH1-1*F* | GGTGTAGGACCGCATAAGGA | Succinate dehydrogenase |
| SDH1-1*R* | GAACCGTGGGTAAGACAGGA | Succinate dehydrogenase |
| SDH1-2*F* | TATTCGCTGGTGTCGATGTT | Succinate dehydrogenase |
| SDH1-2*R* | TCTCCTGCAGCCATTAGTCC | Succinate dehydrogenase |
| SL1*F* | TCAAACCTGGTGAATGCAAG | Succinate CoA ligase |
| SL1*R* | ATTAAACGGATCCCCACCA | Succinate CoA ligase |
| SL2*F* | AACGTGATGAGGCTGAAGAGA | Succinate CoA ligase |
| SL2*R* | CGGTCCAGAATAATGGAAAAG | Succinate CoA ligase |
| SL3*F* | GCAGGAGTGACACCGAAGA | Succinate CoA ligase |
| SL3*R* | CTCAGCCTCAATACCCTCCA | Succinate CoA ligase |
| SSDH*F* | TAGCTCCCCTTATTCGGTTC | Succinic |

| | | |
|---|---|---|
| | | semialdehyde dehydrogenase |
| SSDH*R* | CGTTCACCCCTACAAGTCCA | Succinic semialdehyde dehydrogenase |
| ACT2*F* | CTGGATCGGTGGTTCCATTC | Actin 2 |
| ACT2*R* | CCTGGACCTGCCTCATCATAC | Actin 2 |
| UBQ10*F* | GGCCTTGTATAATCCCTGATGAATAAG | Ubiquitin 10 |
| UBQ10*R* | AAAGAGATAACAGGAACGGAAACATAGT | Ubiquitin 10 |

**Table S1**. Primers used for qRT-PCR.
